# Predictors of change in functional health status in adults with repaired tetralogy of Fallot

**DOI:** 10.1186/1532-429X-18-S1-P166

**Published:** 2016-01-27

**Authors:** Jimmy C Lu, Sunkyung Yu, Ray Lowery, Janaki Sagi, Amanda DeLong, Prachi P Agarwal, Maryam Ghadimi Mahani, Adam L Dorfman

**Affiliations:** 1 grid.214458.e0000000086837370Pediatrics and Communicable Diseases, University of Michigan, Ann Arbor, MI USA; 2 grid.214458.e0000000086837370Radiology, University of Michigan, Ann Arbor, MI USA

## Background

Left ventricular (LV) and right ventricular (RV) ejection fraction (EF) by cardiovascular magnetic resonance (CMR) have been related to functional health status in patients with repaired tetralogy of Fallot (TOF) in cross-sectional studies. However, few longitudinal data are available. This study aimed to determine predictors of subsequent decrease in functional health status in mid-term follow-up in this population.

## Methods

Patients with repaired TOF who had previously completed CMR and assessment with the Short Form 36 version 2 (SF-36) were recruited for repeat CMR, SF-36, and exercise test, if they had not had interval pulmonary valve replacement (PVR). Patients from the same cohort who had undergone PVR were recruited for repeat SF-36 for comparison. LV longitudinal and circumferential strain and RV longitudinal strain were measured using feature tracking software (TomTec, Unterschleissheim, Germany). Functional health status was assessed by age-adjusted z-scores for the SF-36 Physical Component Summary score and subscales of Physical Functioning, Role-Physical, and General Health.

## Results

A total of 19 patients (median 33.5 years old, interquartile range [IQR] 26-42 years, 53% male) had not undergone PVR and were enrolled a median of 5.0 years (IQR 4.8-5.3) since prior CMR and SF-36. LVEF (53.6 ± 5.4 vs. 54.3 ± 6.6%, p = 0.62) and RVEF (47.2 ± 6.4 vs. 46.6 ± 7.0%, p = 0.61) were not significantly different from prior study, while RV end-diastolic volume increased (138 ± 34 vs. 126 ± 31 ml/m^2^, p = 0.02). SF-36 scores also remained stable. General Health z-score correlated with endurance on exercise test (r = 0.52, p = 0.04). Among parameters at the time of initial CMR, higher RV end-systolic volume correlated with decrease in General Health (r= -0.49, p = 0.03); higher pulmonary regurgitant fraction correlated with decrease in Physical Functioning (r= -0.47, p = 0.04) and Role-Physical (r= -0.47, p = 0.04). LVEF, RVEF, strain measurements, and RV end-diastolic volume did not correlate with change in SF-36 scores. In 8 patients who had undergone PVR (median 31.5 years old, IQR 25-44, 50% male), there was an increase in Physical Functioning compared to those without PVR (change in z-score +0.50 ± 0.54 vs. -0.26 ± 0.72, p = 0.01).

## Conclusions

In adults with repaired TOF who do not undergo PVR, LVEF, RVEF and functional health status remain stable in mid-term follow-up. However, RV end-systolic volume at time of CMR inversely correlates with subsequent change in functional health status, underscoring its importance in prognostication and timing of intervention in this population. Although the small sample size limits clinical application, these data, if confirmed in larger cohorts, suggest benefit of PVR in selected patients.

